# Comparison of Outcomes of Non-diffractive Extended Depth of Focus Intraocular Lens and Diffractive Extended Depth of Focus Intraocular Lens

**DOI:** 10.7759/cureus.91413

**Published:** 2025-09-01

**Authors:** Shweta J Vora, Alexander Ebenezar, B Chaitanya, Bharati R Jingar

**Affiliations:** 1 Ophthalmology, Pandit Dindayal Upadhyay Medical College and Hospital, Rajkot, IND; 2 Ophthalmology, Sankara Nethralaya Eye Hospital, Chennai, IND; 3 Ophthalmology, Sankara Eye Institute, Pammal, IND; 4 Ophthalmology, Kolkata Medical College and Hospital, Kolkata, IND; 5 Ophthalmology, ICARE Eye Hospital, Noida, IND

**Keywords:** cataract surgery, contrast sensitivity, extended depth of focus, intermediate vision, near vision, patient satisfaction, symfony intraocular lens, visual acuity outcomes, visual disturbances, vivity intraocular lens

## Abstract

Background

Extended depth of focus (EDOF) intraocular lenses (IOLs) are designed to provide spectacle-free vision across distances. EDOF IOLs can be categorized as diffractive, e.g., Symfony (Abbott Medical Optics, Santa Ana, CA), or non-diffractive, e.g., Vivity (Alcon Inc., Geneva, Switzerland). Non-diffractive and diffractive EDOF technologies offer different approaches to extending the range of focus, with varying effects on visual quality, including glare, halos, and contrast sensitivity.

Aim

To compare visual outcomes between non-diffractive and diffractive EDOF intraocular lenses.

Methodology

This prospective, interventional, randomized, double-arm, comparative study was conducted at a tertiary eye care center in North India, from May 2022 to December 2022. Eighty eyes of 40 patients were enrolled and divided into two groups (Symfony vs. Vivity EDOF lenses). Patients aged 40-80 years with bilateral age-related cataract were included. Standardized phacoemulsification was performed by an experienced surgeon. Visual acuity (distance, intermediate, near) and contrast sensitivity were measured objectively. Glare sensitivity, halos, and patient satisfaction were assessed separately using subjective responses in terms of yes/no. All evaluations were carried out during the one-month postoperative follow-up.

Results

The Mann-Whitney U test was used to compare the Symfony and Vivity groups, each consisting of 40 patients. Before surgery, both the Vivity and Symfony EDOF IOL groups had similar uncorrected vision for distance and near. After surgery, patients in both groups showed clear improvement in uncorrected vision at all distances, i.e., near, intermediate, and far. When comparing the two groups on day one, day seven, and day 30 after surgery, there were no significant differences in visual acuity outcomes, as all p-values for distance, intermediate, and near vision were greater than 0.05.

Contrast sensitivity was nearly the same in both groups on days one, seven, and 30 after surgery. There was no significant difference between the two groups at any time. Both groups had high satisfaction rates, with 38 (95%) patients in each group saying they were satisfied with their surgery. Only two (5%) patients in each group were not satisfied. Vivity EDOF lenses gave better results in terms of glare than Symfony EDOF lenses on all postoperative days (days one, seven, and 30), with statistically significant results.

Conclusions

Our study found no significant differences between Symfony and Vivity IOLs in visual outcomes, contrast sensitivity, or spectacle independence; however, Vivity demonstrated slightly better glare acuity.

## Introduction

Cataract is the leading cause of preventable blindness worldwide. Surgery is the only treatment currently available, although therapeutic and pharmacological treatments are being studied [1,2]. Cataract surgery is rapidly being transformed into a precise refractive surgical technique. There has been a rapid and extensive development of new advanced intraocular lens (IOL) designs in recent years. IOL implants restore optical focusing power, which is lost by the removal of the clouded natural crystalline lens. The devices can be classified as monofocal, multifocal, accommodative, and extended depth of focus (EDOF) IOLs [3,4].

Monofocal IOLs are designed for clear distance vision. With monovision correction, the visual requirements for near with monofocal IOLs can be met; however, this may cause some level of suppression and loss of stereopsis, which ultimately compromises the ultimate binocular visual outcome [3]. Monofocal IOLs can also provide good uncorrected distant and near visual acuity due to favorable corneal wavefront aberrations, favorable corneal astigmatism, or myopic undercorrection. This phenomenon is called pseudophakic monovision [5,6].

“Presbyopia-correcting intraocular lenses” are the solution to this problem, which include multifocal (bifocal and trifocal), accommodative, and EDOF IOLs [3,4]. Bifocal IOLs provide successful outcomes for distant and near vision, although the intended level of improvement cannot be obtained for intermediate vision, which resulted in the introduction of trifocal IOLs.

Trifocal IOLs, which have three focal spots, have been introduced to overcome the problems experienced with bifocal lenses. It has been reported that the use of trifocal IOLs significantly improves intermediate visual acuity (VA) without impairing near and distance vision [7,8].

Multifocal IOLs offer different focusing distances for near and intermediate vision, but are associated with dysphoric symptoms, such as haloes and glare, with reduced mesopic and scotopic contrast sensitivity [3]. Multifocal IOLs provide better uncorrected near visual acuity than monofocal IOLs, leading to less need for spectacles [9]. When compared to monofocal IOLs, multifocal IOLs had statistically significantly better-pooled results for near vision and spectacle dependence, and borderline significantly better quality of vision [10]. Many clinical trials show a higher incidence of positive dysphotopsia (halos and glares) with multifocal IOLs than monofocal IOLs [11,12].

Accommodative IOL designs change the optical power of the eye by either forward or backward axial movement of the IOL or flexibility in lens thickness or shape, and by this, they provide good vision at various distances. However, these lenses are mostly out of clinical practice due to their poor long-term visual outcomes and associated posterior capsular opacification (PCO) and capsular contraction, which leads to asymmetric vaulting and lens tilt [3,4].

Therefore, unmet medical need for presbyopia-correcting IOLs, which are easy to use and have a visual disturbance profile that is comparable with monofocal IOLs, arises [4]. The ideal EDOF lens causes the light to converge to a line, with one end of the line on the retina for distant objects. While viewing near objects, the focal point changes again and shifts; the other end of the line now falls on the retina [13]. They work on the principle of creating a single elongated focal line, enhancing the range of vision [5].

In diffractive IOLs, step structure design causes the light to split into distinct foci using diffraction and delivers vision at distance, intermediate, and/or near. A diffractive EDOF IOL, e.g., the TECNIS Symfony® IOL (Abbott Medical Optics, Santa Ana, CA), is designed to split light using a diffractive optic design to create an elongated focus from distance to intermediate [13].

AcrySof IQ Vivity® (Alcon Inc., Geneva, Switzerland) non-diffractive EDOF IOL utilizes X-WAVE technology (wavefront shaping) to create a continuous EDOF effect. The wavefront shaping profile is placed on the anterior surface of the Vivity IOL, which shapes the beam of light entering the eye so that, as it propagates to the retina, the light is mostly confined to the EDOF channel. The Vivity IOL provides a continuous extended range of vision from distance through intermediate and functional near vision, with a low rate of visual disturbances [4,13].

## Materials and methods

Study design

The study was a prospective, interventional, randomized, comparative, double-arm study conducted over a period of eight months, from May 2022 to December 2022. The selection criteria are presented in Table 1.

**Table 1:** Selection criteria for study population.

Criteria type	Details
Inclusion criteria	Age between 40 and 80 years
	Bilateral age-related cataract
	Eligible for bilateral intraocular lens implantation within one month
	No history of ocular or refractive surgery
	Provided written informed consent to participate in the study
	Corneal astigmatism, if present, should be less than 1D
Exclusion criteria	Significant anterior segment pathology like corneal opacity, severe dry eye, uveitis, glaucoma, pseudoexfoliation, etc.
	Any obvious posterior segment pathology, like retinopathy or macular degeneration
	Corneal astigmatism more than 1D
	Patient with inadequate pupil dilation
	High myopia (axial length > 25 mm)
	History of previous eye surgery
	Any intraoperative and postoperative complications
	Uncontrolled systemic diseases (e.g., diabetes and hypertension)
	Were lost to follow-up during the study period

Methodology

Preoperative Evaluation

All patients underwent a comprehensive preoperative evaluation that included a detailed clinical history, complete systemic examination, and thorough ocular assessment. Visual acuity was measured for distance (4 m), intermediate (66 cm), and near (33 cm), both uncorrected and best-corrected. These values were converted to logMAR units under photopic conditions (85 cd/m²) using the Early Treatment Diabetic Retinopathy Study (ETDRS) chart. The refractive status of the eye was assessed with streak retinoscopy, followed by a complete slit-lamp biomicroscopic examination. A dilated fundus evaluation was performed using indirect ophthalmoscopy with 20D and 90D lenses. Biometric measurements were obtained, including axial length using the IOLMaster 700 (ZEISS, Baden-Württemberg, Germany). In addition, IOL power was calculated with the IOLMaster 700 employing the Barrett formula.

Surgical Technique

Phacoemulsification cataract surgery was done with the implantation of diffractive and non-diffractive EDOF lenses by a single surgeon.

Postoperative Evaluation

Uncorrected visual acuity for distance (4 m), intermediate (66 cm), and near (33 cm) was assessed at one and seven days, while both uncorrected and best-corrected visual acuity were evaluated at 30 days. All measurements were recorded in logMAR units under photopic conditions (85 cd/m²) using the ETDRS chart positioned at 4 m. Contrast sensitivity was measured with the Pelli-Robson chart, which presents letters of uniform size subtending 5° of visual angle at 3 m, with logCS values increasing in 0.15 steps. Glare disability was assessed by inducing glare with a disposable pen torch at the pupillary margin and recording visual acuity on the ETDRS chart. In addition, slit-lamp evaluation, intraocular pressure measurement with applanation tonometry, and dilated fundus examination with a 20D lens were performed. At 30 days, a subjective assessment of visual quality was obtained using a post-cataract surgery questionnaire.

Endpoints

Primary Outcome

Assessment of the success rate in providing spectacle independence with the help of diffractive and non-diffractive EDOF IOLs.

Secondary Outcome

Assessment of patient satisfaction in terms of halos, glare, and contrast sensitivity.

Sampling

The primary outcome was uncorrected intermediate visual acuity (UCIVA, logMAR) at 66 cm under photopic illumination (≈85 cd/m²). The study was powered to detect a clinically meaningful difference of 0.1 logMAR with an assumed standard deviation of 0.15 (based on pilot data from our setup). With α = 0.05 (two-sided) and 80% power, the required sample size was 36 eyes per group. Allowing for a 10% loss to follow-up, the final target sample was 40 eyes per group (80 eyes in total).

Randomization

Block randomization was done using sealed envelopes. Each block contained 10 envelopes: five for group A (Vivity IOL) and five for group B (Symfony IOL). Patients were assigned by opening one envelope after obtaining consent.

Data analysis

Categorical variables were presented as numbers and percentages (%), and continuous variables were presented as mean ± SD and median. The normality of data was tested using the Kolmogorov-Smirnov test. If normality was rejected, nonparametric tests were used.

Statistical tests were applied as follows: quantitative variables were compared using the unpaired t-test or Mann-Whitney test (when the datasets were not normally distributed) between the two groups.

A p-value of <0.05 was considered statistically significant. The data were entered into a Microsoft Excel spreadsheet (Microsoft Corporation, Redmond, WA), and analysis was performed using SPSS version 21.0 (IBM Corp., Armonk, NY).

## Results

The mean age of the patients in the Symfony group was 63.6 ± 7.03 years, while the mean age of the patients in the Vivity group was 64.6 ± 6.14 years. In the Symfony group, 16 (40%) patients were female, and 24 (60%) patients were male. In the Vivity group, 12 (30%) patients were female and 28 (70%) were male.

The baseline characteristics of the two groups were comparable. The mean preoperative uncorrected distant visual acuity (UCDVA) was 1.14 ± 0.44 logMAR in the Symfony group and 1.14 ± 0.45 logMAR in the Vivity group, with no statistically significant difference (p = 0.496). Similarly, the mean uncorrected near visual acuity (UCNVA) was 0.56 ± 0.11 logMAR in the Symfony group and 0.57 ± 0.10 logMAR in the Vivity group (p = 0.333). The mean uncorrected intermediate visual acuity (UCIVA) was 0.56 ± 0.97 logMAR in the Symfony group and 0.57 ± 0.09 logMAR in the Vivity group, also showing no significant difference (p = 0.366).

Corneal keratometry values were comparable between the two groups. The mean K1 (horizontal keratometry) was 44.18 ± 1.11 D in the Symfony group and 44.49 ± 0.87 D in the Vivity group (p = 0.845). The mean K2 (vertical keratometry) was 44.62 ± 1.15 D and 44.79 ± 0.94 D, respectively (p = 0.239).

The mean axial length was 23.37 ± 0.87 mm in the Symfony group and 23.16 ± 0.77 mm in the Vivity group, with no statistically significant difference (p = 0.129). The mean IOL power implanted was 21.76 ± 1.96 D in the Symfony group and 22.18 ± 2.08 D in the Vivity group, which was also comparable (p = 0.178).

Comparison of UCDVA at postoperative days one, seven, and 30

Because the data were non-parametric, the Mann-Whitney U test was used to compare the two groups, each consisting of 40 patients. Patients in both the Vivity and Symfony EDOF IOL groups showed clear improvement in distance vision after surgery. The average vision improved significantly after surgery, dropping from 1.14 logMAR before the procedure to approximately 0.07 logMAR on day one in both the Symfony and Vivity groups. On day seven, the vision was 0.07 in the Symfony group and 0.06 in the Vivity group. By day 30, it improved further to 0.06 for Symfony and 0.05 for Vivity.

**Figure 1:**
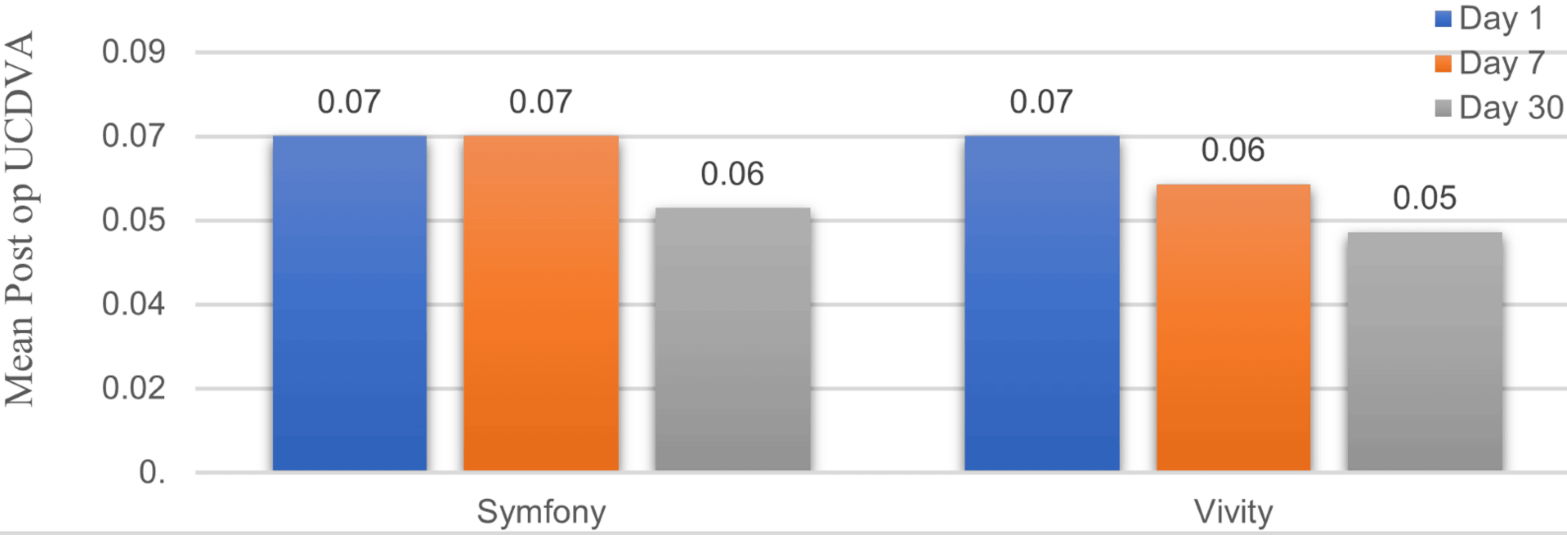
Comparison of mean postoperative uncorrected distant visual acuity (UCDVA) (N = 40 in each group).

This difference was not statistically significant between both groups (p-values: 0.488, 0.250, and 0.426). All effect sizes are small (0.077, 0.129, and 0.089), indicating minimal practical difference between the Symfony and Vivity groups, consistent with the non-significant p-values (Figure 1).

Comparison of best corrected distance visual acuity on day 30

After applying the Mann-Whitney U test to compare 40 patients in each group, it was found that the mean best corrected distance visual acuity (BCDVA) on day 30 was 0.04 ± 0.048 logMAR in the Symfony group and 0.03 ± 0.045 logMAR in the Vivity group. This difference was not statistically significant (p = 0.2377), indicating that both IOLs provided comparable visual outcomes at one month postoperatively. There is a small effect size (0.13), suggesting a minimal difference in BCDVA between the Symfony and Vivity IOL groups on day 30, consistent with the non-significant p-value.

Comparison of UCIVA at postoperative days one, seven, and 30

**Figure 2:**
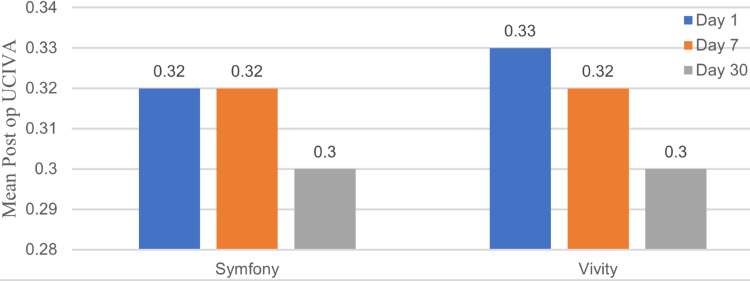
Comparison of mean postoperative uncorrected intermediate visual acuity (UCIVA) (N = 40 in each group).

The Mann-Whitney U test was used to compare the Symfony and Vivity groups, each consisting of 40 patients. The mean UCIVA values were comparable between the Symfony and Vivity groups at all time points. On postoperative day one, mean UCIVA was 0.32 logMAR for Symfony and 0.33 for Vivity (p = 0.310). By day seven, mean UCIVA was 0.32 logMAR for both Symfony and Vivity (p = 0.500), and at day 30, both improved slightly to 0.30 logMAR (p = 0.0779). All effect sizes are small (0.114, 0.075, and 0.196), with day 30 approaching small-to-moderate (0.196), suggesting minimal practical difference in UCIVA between Symfony and Vivity at all follow-up points (Figure 2).

Comparison of best corrected intermediate visual acuity on day 30

The mean best corrected intermediate visual acuity (BCIVA) on day 30 for the Symfony group and the Vivity group was 0.3 ± 0.00 logMAR and 0.3 ± 0.00 logMAR, respectively. There was no statistically significant difference between the two groups (p = 0.5), as determined by the Mann-Whitney U test applied due to the non-parametric nature of the data. A very small effect size (0.075) was noted, confirming the lack of meaningful difference in BCIVA between the Symfony and Vivity groups at day 30.

Comparison of UCNVA at postoperative days one, seven, and 30

**Figure 3:**
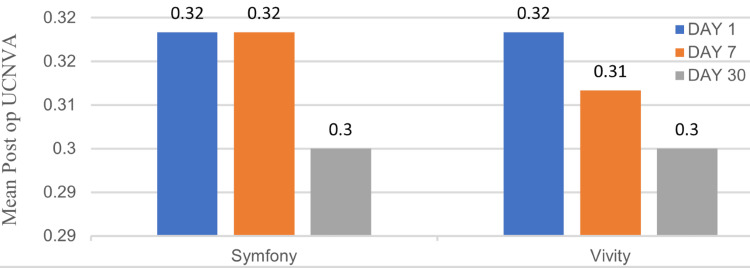
Comparison of mean postoperative uncorrected near visual acuity (UCNVA) (N = 40 in each group).

Both groups had similar near vision before surgery (Mann-Whitney U test, N = 40 in each group). Both groups showed clear improvement in near vision after surgery. After surgery, there was no significant difference between the Symfony and Vivity groups at day one (mean UCNVA: 0.32 vs. 0.32 logMAR, p = 0.5), day seven (mean UCNVA: 0.32 vs. 0.31 logMAR, p = 0.1077), and day 30 (mean UCNVA: 0.3 vs. 0.3 logMAR, p = 0.5). All comparisons show small or very small effect sizes (0.075, 0.180, and 0.075), consistent with the non-significant p-values (Figure 3).

Comparison of best corrected near visual acuity at day 30

After applying the Mann-Whitney U test to compare 40 patients in each group, it was found that the mean best corrected near visual acuity (BCNVA) was 0.3 ± 0.00 log MAR in the Symfony group and 0.3 ± 0.02 log MAR in the Vivity group. There was no statistically significant diﬀerence. A very small effect size (0.075) was noted, supporting the conclusion that both Symfony and Vivity IOLs resulted in comparable BCNVA postoperatively.

Comparison of postoperative contrast sensitivity at postoperative days one, seven, and 30

The Mann-Whitney U test was applied to compare 40 patients in each group. On postoperative day one, both groups had identical mean contrast sensitivity scores (1.78 vs. 1.78 logMAR; p = 0.5). Similarly, on day seven, the mean contrast sensitivity remained the same in both groups (1.78 vs. 1.78 logMAR; p = 0.5). By day 30, there was a slight improvement in both groups, with the Symfony group showing a mean contrast sensitivity of 1.835 logMAR and the Vivity group showing 1.85 logMAR; however, this difference was not statistically significant (p = 0.077). On days one and seven, the effect size was very small (0.075, 0.075), confirming no meaningful difference. By day 30, a slightly larger effect (0.20) was noted, but still small and not statistically significant (Table 2).

**Table 2:** Comparison of postoperative mean contrast sensitivity (N = 40 in each group).

Postoperative day (POD)	Symfony	Vivity	p-value
POD 1	1.78	1.78	0.5
POD 7	1.78	1.78	0.5
POD 30	1.835	1.85	0.077

Comparison of glare acuity at postoperative days one, seven, and 30

On the first and seventh days after surgery, patients in the Symfony group experienced more glare than those in the Vivity group, as assessed using the Mann-Whitney U test due to non-parametric data. The average glare score was 0.12 logMAR for the Symfony group and 0.04 logMAR for the Vivity group, and this difference was statistically significant (p = 0.0087). Even by day 30 after surgery, the Symfony group still had slightly more glare (0.12 vs. 0.03 logMAR), and this difference remained statistically significant (p = 0.029).

On postoperative days one and seven, patients in the Symfony group experienced significantly more glare than those in the Vivity group, with a moderate effect size (0.29, 0.29). This difference remained statistically significant on day 30, though the effect size was slightly smaller (0.24), indicating a small to moderate effect. These findings suggest that the Vivity group had better glare tolerance compared to the Symfony group throughout the postoperative period (Table 3).

**Table 3:** Comparison of mean postoperative glare score (N = 40 in each group).

Postoperative day (POD)	Symfony	Vivity	p-value
POD 1	0.12	0.04	0.0087
POD 7	0.12	0.04	0.0087
POD 30	0.12	0.13	0.029

Comparison of overall visual satisfaction between the Symfony and Vivity groups in terms of subjective response of yes or no

A total of 38 (95%) patients of both the Symfony and Vivity groups were satisfied with surgery, while two (5%) patients of the Symfony and Vivity groups were not satisfied with their surgery. There was no statistically significant difference between the two groups (Table 4).

**Table 4:** Comparison of overall visual satisfaction between the Symfony and Vivity groups (N = 40 each group).

Overall visual satisfaction	Symfony	Vivity	Total	p-value
Yes	38 (95%)	38 (95%)	76 (95%)	1.0
No	2 (5%)	2 (5%)	4 (5%)	
Total	40 (100%)	40 (100%)	80 (100%)	

Comparison of spectacle independence for distant, intermediate, and near visual acuity according to subjective response on postoperative day 30

In the Symfony group, spectacle independence for distance, intermediate, and near vision was achieved in 38 (95%), 40 (100%), and 38 (95%) patients, respectively, at day 30.

In the Vivity group, spectacle independence for distant, intermediate, and near vision was achieved in 38 (95%), 40 (100%), and 38 (95%) patients, respectively, at day 30.

There was no statistically significant difference between the two groups for the requirement of glasses for distant, intermediate, and near distances.

Comparison of refraction on postoperative day 30

There was no statistically significant difference in refractive error between the two groups at day 30 (p > 0.05, Mann-Whitney U test).

## Discussion

**Figure 4:**
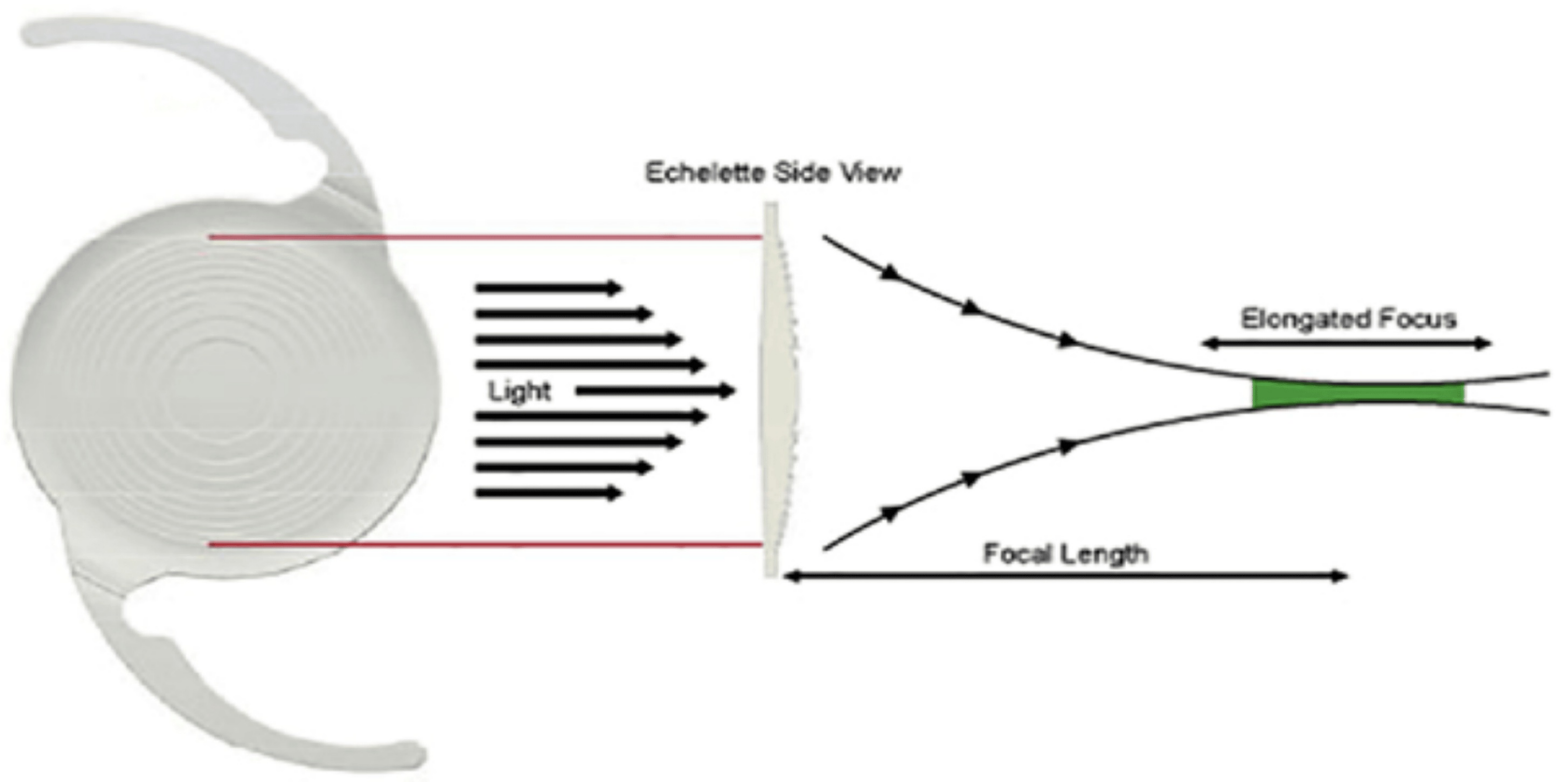
Design and mechanism of action of diffractive extended depth of focus intraocular lens (Symfony). Source: Cochener (2016) [14]. This figure has been used with permission under license obtained from the publisher.

EDOF IOLs are designed to provide spectacle-free vision across distances. EDOF IOLs can be categorized as diffractive (e.g., Symfony) or non-diffractive (e.g., Vivity). Diffractive IOLs, such as the TECNIS Symfony® IOL, use a step-like diffractive optic design that splits light to create an elongated focus. This provides useful vision from distance to intermediate but may increase the risk of photic phenomena such as glare and halos (Figure 4) [13,14].

**Figure 5:**
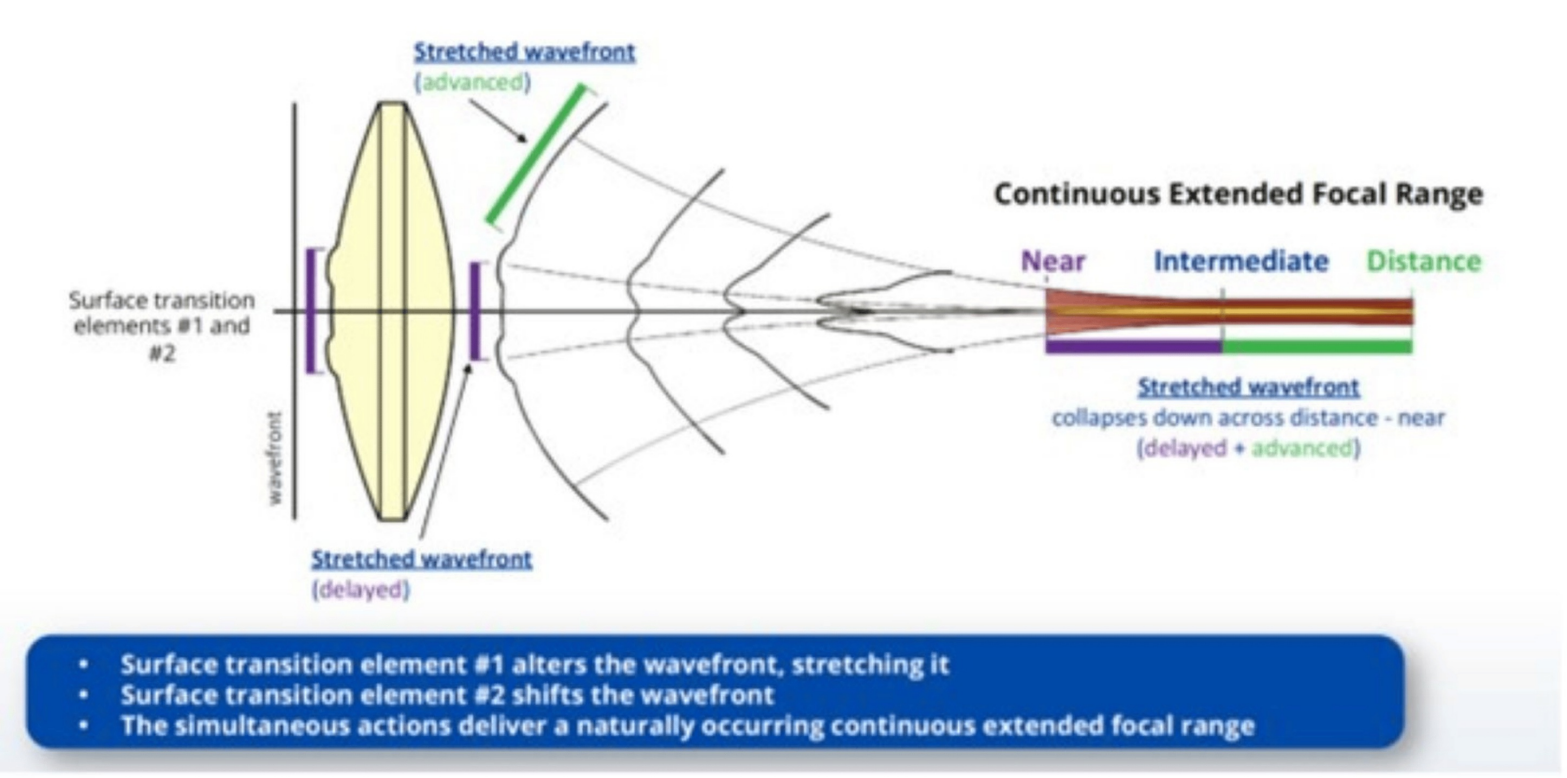
The Vivity intraocular lens utilizes the X-WAVE technology. Source: Alcon. Optical principles of extended depth of focus IOLs. (2025) [13]. This figure is adapted from an open-access journal article.

In contrast, the AcrySof IQ Vivity® IOL is a non-diffractive EDOF lens. It employs X-WAVE™ technology to reshape the wavefront rather than splitting light, thereby creating a smooth and continuous range of vision from distance through intermediate, with functional near vision. This non-diffractive design is associated with fewer visual disturbances compared to diffractive IOLs (Figure 5) [4,13,14].

The mean UCDVA for the Symfony and Vivity groups on first, seventh, and 30th postoperative days was not statistically significant, with p-values of 0.488, 0.250, and 0.426, respectively. There was no statistically significant difference between the BCDVA at day 30, with p = 0.237. Similar to our study, Song et al. [15] also found no significant difference in UCDVA of the two groups, with p = 0.717.

In our study, there was no statistically significant difference in mean UCIVA between the Symfony and Vivity groups on postoperative days one, seven, and 30, with p-values of 0.310, 0.500, and 0.077, respectively. There was no statistically significant difference between the BCIVA of the two groups at day 30, with a p-value of 0.5. Our findings are in line with those of Song et al. [15], who also reported no significant difference in UCIVA between the two groups, with p-values of 0.717. The BCIVA p-value was 0.468, which was not statistically significant; therefore, the results were in agreement with our study.

UCNVA did not differ significantly between the Symfony and Vivity groups at postoperative days one, seven, and 30 (p = 0.5, 0.107, and 0.5, respectively). These results contrast with the findings of Song et al. [15], who observed a significant difference in UCNVA between the two groups, with the Symfony group showing superior UCNVA.

There were no statistically significant differences in contrast sensitivity between the Symfony and Vivity groups at postoperative days one, seven, and 30, with p-values greater than 0.05. However, due to the lack of standardized contrast sensitivity tests across studies, comparing our findings to those from other research remains difficult.

There was a statistically significant difference in glare acuity at postoperative days one, seven, and 30 for the Symfony group as compared to the Vivity group, with p-values of 0.008, 0.008, and 0.029, respectively. The Vivity group had better glare acuity than the Symfony group at day one, seven, and 30. However, the lack of comparative data between the glare acuity tests in different studies makes it difficult to directly compare the outcomes of different studies.

By day 30, both the Symfony and Vivity groups showed high spectacle independence, 95% for distance and near, and 100% for intermediate vision. There was no statistically significant difference between the two groups in terms of glasses requirement at any distance. These findings align with those of Song et al. [15], who also reported no significant difference in spectacle independence across visual ranges.

Van Amelsfort et al. (2022) reported that with AcrySof IQ Vivity in mini monovision, 96% of patients rarely needed glasses for distance, 68% for intermediate, and 38% for near vision, indicating strong performance at distance and intermediate ranges but limited near vision [16]. In a multicenter study, Coassin et al. found that the AcrySof IQ Vivity lens, when used with mini monovision, led to complete spectacle independence in 87% of patients [17].

In our study, in both the Symfony and Vivity groups, 95% of patients (38 out of 40 in each group) reported being satisfied with their overall vision after surgery. Only two patients in each group (5%) were not satisfied. These findings are in line with the prospective study by van Amelsfort et al. (2022), which evaluated AcrySof IQ Vivity with mini monovision and reported minimal visual disturbances; 91% of patients experienced no halos or glare, and 100% reported no starbursts, indicating excellent visual quality and patient comfort [16].

Limitations of the study

This study has several limitations. First, the sample size was small and the follow-up period was only 30 days, preventing evaluation of long-term outcomes. Second, important refractive details, such as preoperative refractive error and target refraction, were not reported, and there was a lack of adjustment for paired-eye analysis. Third, functional assessments such as glare and visual satisfaction were not performed using standardized or validated techniques, which may have introduced bias and restricted comparison with other studies. Finally, the single-center design reduces the external validity of our results. Larger multicenter trials with extended follow-up and standardized outcome measures are required to address these limitations.

## Conclusions

Our study found no statistically significant differences between Symfony and Vivity IOLs in terms of uncorrected and best corrected distance, intermediate, and near visual acuity on postoperative days one, seven, and 30. Both lens types also showed comparable performance in contrast sensitivity and spectacle independence across all visual ranges. Although the Vivity group demonstrated slightly better glare acuity. Overall, both lens types were effective in restoring functional vision across various distances and lighting conditions.
